# History of Hypertension Is Associated With MR Hypoperfusion in Chinese Inpatients With DWI-Negative TIA

**DOI:** 10.3389/fneur.2019.00867

**Published:** 2019-08-14

**Authors:** Yue Wang, Huazheng Liang, Yu Luo, Yuan Zhou, Lingjing Jin, Shaoshi Wang, Yong Bi

**Affiliations:** ^1^Department of Neurology, Shanghai Fourth People's Hospital Affiliated to Tongji University School of Medicine, Shanghai, China; ^2^Department of Neurology, Translational Research Institute of Brain and Brain-Like Intelligence, Shanghai Fourth People's Hospital Affiliated to Tongji University School of Medicine, Shanghai, China; ^3^Department of Radiology, Shanghai Fourth People's Hospital Affiliated to Tongji University School of Medicine, Shanghai, China; ^4^Department of Neurology, Tongji Hospital, Tongji University, Shanghai, China

**Keywords:** DWI, PWI, transient ischemic attack, risk factors, hypertension

## Abstract

**Objectives:** The present study aimed to examine the prevalence of and risk factors for magnetic resonance (MR) perfusion abnormality in a Chinese population with transient ischemic attack (TIA) and normal diffusion-weighted imaging (DWI) findings.

**Methods:** Patients with TIA admitted to our stroke center between January 2015 and October 2017 were recruited to the present study. MRI, including both DWI and perfusion-weighted imaging (PWI), was performed within 7 days of symptom onset. Time to maximum of the residue function (T_max_) maps were evaluated using the RAPID software (Ischemaview USA, Version 4.9) to determine hypoperfusion. Multivariate analysis was used to assess perfusion findings, clinical variables, medical history, cardio-metabolic, and the ABCD2 scores (age, blood pressure, clinical features, symptom duration, and diabetes).

**Results:** Fifty-nine patients met the inclusion criteria. The prevalence of MR perfusion T_max_ ≥ 4 s ≥ 0 ml and ≥ 10 mL were 72.9% (43/59) and 42.4% (25/59), respectively. Multivariate analyses revealed that history of hypertension is an independent factor associated with MR perfusion abnormality (T_max_ ≥ 4 s ≥ 10 mL) for Chinese patients with TIA (*P* = 0.033, adjusted OR = 4.11, 95% CI = 1.12–15.11). Proximal artery stenosis (>50%) tended to lead to a larger PW lesion on MRI (*p* = 0.067, adjusted OR = 3.60, 95% CI = 0.91–14.20).

**Conclusion:** Our results suggest that the prevalence of perfusion abnormality is high as assessed by RAPID using the parametric T_max_ ≥ 4 s. History of hypertension is a strong predictor of focal perfusion abnormality as calculated by RAPID on T_max_ map of TIA patients with negative DWI findings.

## Introduction

Transient ischemic attack (TIA) has been redefined as a transient episode of neurological dysfunction caused by focal brain, spinal cord, or retinal ischemia without evidence of acute infarction (1). According to a multicenter, community-based study, the population of TIA survivors at any given time in China is as large as 10–12 million (2). TIA is associated with high risk of early subsequent stroke up to 20% of patients (3). TIA has been evaluated as a major risk factor for future recurrent ischemic attacks, and emergent diagnosis of the cause is needed to ensure timely treatment and to dramatically reduce the risk of developing strokes (4–6).

Prognosis of TIA depends on not only its pathological basis, but also early identification of high-risk patients with TIA and timely treatment. Usually, TIA diagnosis relies primarily on the reported history. The ABCD2 prediction score (range 0–7, age, blood pressure, clinical symptoms, duration, and diabetes) was originally intended to aid non-specialists in community and emergency department settings to improve risk stratification of patients with transient neurological symptoms, and had little specificity between hospital-based neurologists (7). Therefore, the diagnosis of TIA based on symptoms alone is challenging (8). Moreover, agreement on the vascular origin of transient neurologic symptoms can be low, even among experienced neurologists (9, 10). Early evaluation using imaging techniques is essential for administering the proper medications to treat or prevent TIA and the consequent stroke, which will refine the clinical diagnosis of TIA.

Based on the current diagnostic criteria, TIA is defined as a condition in which transient episode of neurological dysfunction exists without lesions on DWI. However, imaging results of TIA patients show diverse pictures. For example, perfusion-weighted imaging (PWI) shows either positive or negative findings in DWI negative patients. It is estimated that 23–42% of patients with TIA who have a negative DWI show PWI positive lesions (11–15). Acute PWI abnormality is associated with recurrent attacks and even infarct progression (13, 15–18). Therefore, low perfusion may be one of the pathological mechanisms of TIA recurrence. However, little research has been done on the relationship between TIA with negative DWI and perfusion abnormality in Chinese populations. The aim of the present study, therefore, was to assess the prevalence of MR perfusion abnormality and its risk factors in Chinese patients with TIA and negative DWI.

## Methods

### Subjects

We retrospectively identified patients with TIA admitted to our stroke center between January 2015 and October 2017. The inclusion criteria for this study: (a) patients presented with TIA and evaluated by a certified stroke neurologist at the time of admission and discharge, diagnosis of TIA was confirmed by two certified stroke neurologists; (b) MRI including both DWI and PWI within 7 days of symptom onset, and no DWI evidence of restricted diffusion; (c) Time to maximum of the residue function (T_max_) maps were assessed independently using the RAPID software (Ischemaview USA, Version 4.9). The exclusion criteria: (a) patients with TIA did not have perfusion status assessed, or had DWI showing a lesion; (b) Patients received revascularization therapy (thrombolysis/thrombectomy). Radiologists blinded to clinical information independently evaluated the presence of acute ischemic lesions detected on DWI/PWI. Demographic data, clinical variables, risk factors, ABCD2 scores, neurologic deficits, duration of TIA, number of TIA attacks, time between MRI and onset were documented for each patient. Ethical approval for this study (2018011) was obtained from Human Research Ethics Committee of Shanghai Fourth People's Hospital Affiliated to Tongji University School of Medicine.

### Imaging

MRI was performed using a 1.5-T Avanto scanner (Siemens, Erlangen, Germany). The imaging protocol included DWI, FLAIR, PWI, and MR angiography (MRA). Imaging parameters were listed below. The head coil is an-8-channel phased-array coil. Axial EPI-DWI: 19 slices, slice thickness = 5.5 mm; TR/TE, 3,600/102 ms; FOV = 230 mm^2^, b = 0 and 1,000 s/mm^2^; EPI factor = 192; matrix = 192 × 192; bandwidth = 964 Hz/pixel. Axial FLAIR: 18 slices, slice thickness = 5.5 mm; TR/TE, 4,000/92 ms; FOV = 230 mm^2^; TI = 1,532.6 ms; Matrix = 256 × 190; bandwidth = 190 Hz/Px; flip angle = 150°. Axial EPI-PWI: 19 slices, slice thickness = 5, 1.5 mm spacing; TR/TE, 1,590/32 ms; measurements = 50; FOV = 230 mm^2^; matrix size = 128 × 128; band width = 1,346 Hz/pixel; flip angle = 90°. Gd-DTPA contrast agent (gadopentetate dimeglumine; Shanghai Pharmaceutical Corporation, Shanghai, China) was intravenously injected (0.2 mmol/kg body weight) at a rate of 4 mL/s after flushing with 30 ml saline. Time-of-flight MR angiography: slice thickness = 0.7 mm; TR/TE, 25/7 ms; FOV = 180 mm^2^; Matrix = 241 × 256; Bandwidth = 100 Hz/PX; flip angle = 25°.

Based on the clinical manifestation of TIA patients, the ischemic lesion site was localized.

Estimates of the volume of hypoperfusion from MRI perfusion scans were performed using the RAPID software, which is an automated image post-processing system (19). We used RAPID in our trial to measure the volume of hypoperfusion (20). Lesion volumes of T_max_ ≥ 4 s were used for determining perfusion deficits in TIA patients with negative DWI findings (13, 15).

### Statistical Analysis

Continuous variables were presented with mean ± standard deviation (SD) or median with interquartile range (IQR); categorical variables were summarized as percentages. The normality of distribution for continuous variables was checked with the one-sample Kolmogorov–Smirnov test. Baseline information of patients with and without MR perfusion abnormality was compared using the independent sample *t*-test or Mann-Whitney *U*-test for continuous variables and Pearson chi-square or Fisher's exact tests for categorical variables. Binary logistic regression was used to assess the independent association between perfusion abnormality and risk factors. Univariate binary logistic regression analysis was used to screen for possible risk factors using *P* < 0.1. We assessed odd ratios (OR) of two patterns of perfusion abnormality for categorical variables (MR perfusion T_max_ ≥ 4 s < 10 ml as no abnormality, and T_max_ ≥ 4 s ≥ 10 ml as abnormality) with MRI perfusion normality being used as the reference. Correlation between TIA patients clinical information and perfusion abnormality with respect to MRI perfusion was tested using the multiple logistic regression analysis modeling with the “Enter” method. The multivariate regression model included history of hypertension and stenosis (50%) with a univariate *P* < 0.1 as independent variables. Meanwhile, the ABCD2 score, which is known to be correlated with perfusion abnormality, was also included for further analysis though its *P* > 0.1.

All association data were expressed as OR with corresponding 95% confidence intervals (CI) and *P*-values. Two-tailed tests were used for all analyses, with the statistical significance level set at 0.05. The data were analyzed with SPSS (version 20.0) for Windows (SPSS Inc., Chicago, IL, USA).

## Results

A total of 154 patients records were evaluated for probable TIA at the Stroke Center of Shanghai Fourth People's Hospital Affiliated to Tongji University School of Medicine between January 2015 and October 2017. Fifty nine patients (24 women, 35 men; age range, 49–86 years; mean, 69 years) met the inclusion criteria. Sixty three patients were excluded because perfusion weighted images were not available (*n* = 63) after a TIA. Another 12 patients were excluded because they were not given a discharge diagnosis of tissue-negative TIA. Eighteen patients had DWI positive lesions, and another two had inadequate information.

### Patient Baseline Characteristics

A total of 59 subjects, including 35 males and 24 females, were included in the study. The median age of patients was 69 [interquartile range (IQR): 63–78]. Median (IQR) ABCD2 score was 4 (2–4). Baseline perfusion scans were performed after a median (IQR) delay of 5 (4–9) days from symptom onset or five (IQR 3–8) days from last attack. The median (IQR) symptom duration was 15 (5–60) min and the median frequency of TIA attacks at baseline was one (IQR 1–2). The average total cholesterol of patients was 4.24 ± 1.15 mmol/L, ranging from 1.94 to 8 mmol/L. The mean low-density lipoprotein (LDL) cholesterol level was 2.22 ± 0.84 mmol/L, ranging from 0.65 to 4.42 mmol/L. The average fasting blood-glucose (FBG) was 5.65 ± 1.40 mmol/L, ranging from 4.4 to 12.2 mmol/L. A history of hypertension was present in 67.8% (40/59) of patients, diabetes mellitus in 27.1% (16/59), and atrial fibrillation in 3.4% (2/59), history of stroke in 28.8% (17/59), smoking in 30.5% (18/59), and anterior circulation symptoms in 54.2% (32/59) (Table 1).

**Table 1:** Basic demographic and clinical characteristics of TIA patients (*n* = 59) stratified according to the presence of MR perfusion abnormality.

**Characteristics**	**Total (*n* = 59)**	**T_**max**_ ≥ 4 s <10 mL (*n* = 34)**	**T_**max**_ ≥ 4 s ≥ 10 mL (*n* = 25)**	**Z, t, or X^**2**^**	***P* value**
Age, y, median (IQR)	69.0 (63.0–78.0)	68.5 (61.75–75.75)	69 (63.5–80.0)	1.154	0.248
Female, *n* (%)	24 (40.7)	14 (41.2)	10 (40.0)	0.051	0.928
**Medical history**, ***n*** **(%)**					
Hypertension	40 (67.8)	19 (55.9)	21 (84.0)	5.217	0.022
Diabetes mellitus	16 (27.1)	10 (29.4)	6 (24.0)	0.213	0.644
Atrial fibrillation	2 (3.4)	0 (0)	2 (8.0)	2.815	0.093
Smoking	18 (30.5)	10 (29.4)	8 (32.0)	0.046	0.831
Drinking	8 (13.6)	6 (17.6)	2 (8.0)	1.144	0.447
Stroke	17 (28.8)	8 (23.5)	9 (36.0)	1.092	0.296
**Cardo-metabolic**					
Ghb (g/L)	129.59 ± 18.17	127.21 ± 19.78	132.84 ± 15.49	1.181	0.243
NEU%	64.07 ± 7.82	64.16 ± 8.88	63.94 ± 6.26	0.110	0.913
FBG (mmol/L)	5.2 (4.9–5.9)	5.20 (4.88–5.95)	5.3 (4.90–5.95)	0.061	0.951
2hBG (mmol/L)	7.64 (6.44–11.7)	7.28 (6.30–12.10)	8.32 (6.64–11.40)	0.629	0.529
Triglyceride (mmol/L)	1.7 (1.15–2.61)	1.62 (1.12–2.78)	1.75 (1.21–2.41)	0.307	0.759
Total cholesterol (mmol/L)	4.24 ± 1.15	4.30 ± 1.14	4.15 ± 1.19	0.494	0.624
LDL (mmol/L)	2.22 ± 0.84	2.27 ± 0.82	2.15 ± 0.88	0.552	0.583
ESR (mm/h)	14 (6–28)	13.5 (6.75–28.25)	18 (6–28)	0.177	0.860
Days_before, day, median (IQR)	2.0 (0.6–5.0)	1.50 (0.64–5.00)	2 (0.52–7.00)	0.131	0.896
Times_before, median (IQR)	1 (1–2)	1 (1–2.25)	1 (1–2)	0.337	0.736
ABCD2 score, median (IQR)	4 (2–4)	4 (2.75–5)	4 (2–4)	0.228	0.819
Duration_onset, min, median (IQR)	15 (5–60)	20 (5–60)	10 (4.00–45.00)	0.819	0.284
sBP at admission, median (IQR)	140 (130–150)	140 (130–142)	140 (130–153)	1.335	0.182
Days_inhos, median (IQR)	10 (9–13)	9 (8.00–13.00)	11 (10–12.5)	1.615	0.106
Perfusion_first, d, median (IQR)	5 (4–9)	5.5 (3.0–9.25)	5 (4.0–8.5)	0.337	0.926
Perfusion_last, d, median (IQR)	5 (3–8)	4.75 (3–8)	5 (4–6)	0.231	0.817
Stenosis (50%) *n* (%)	12 (20.3)	4 (11.8)	8 (32)	3.641	0.056
Anterior *n* (%)	32 (54.2)	18 (52.9)	14 (56.0)	0.231	0.817

*Tmax ≥ 4 s, Time to maximum of the residue function ≥ 4 s; IQR, interquartile range; Ghb, hemoglobin; NEU%, neutrophil percentage; FBG, fasting blood-glucose; LDL, low-density lipoprotein cholesterol; ESR, erythrocyte sedimentation rate; sBP, systolic blood pressure; Days_inhos, Days in hospital*.

*ABCD2: age 60 (1 point), SBP 140, or DBP 90 mm Hg (1 point), clinical features as unilateral weakness (2 points) or speech impairment without weakness (1 point), symptom duration 60 min (2 points), or 10–59 min (1 point), diabetes (1 point)*.

*Significant difference when P <0.05*.

### Comparison of Demographic and Clinical Variables Between Patients With and Without MR Perfusion Abnormality

**Figure 1:**
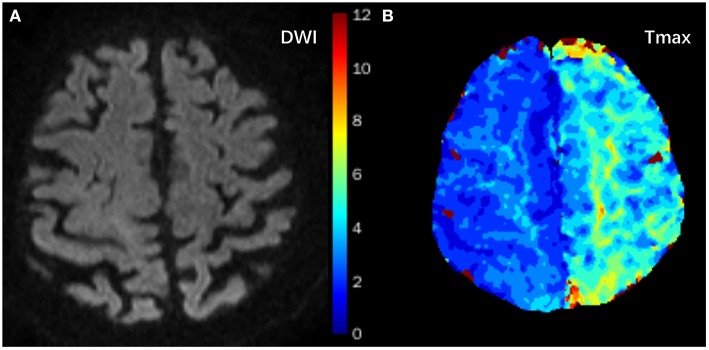
Typical images of an 84-year old female with a history of hypertension who presented with right upper limb paresis three times within 4 h. It lasted 2 min each time. DWI showed negative findings of strokes **(A)**, but T_max_ showed focal hypoperfusion areas in the left frontal and parietal lobes (**B**, green areas).

The prevalence of MR perfusion T_max_ ≥ 4 s > 0 mL and T_max_ ≥ 4 s ≥10 mL was 72.9% (43/59) and 42.4% (25/59), respectively. Figure 1 showed typical images of an 84 year old female whose DWI showed negative findings of strokes, but PWI showed a focal lesion on Tmax.

Table 1 presented the socio-demographic characteristics and clinical risk factors associated with MRI perfusion abnormality. Comparisons of these variables between patients with and without PWI abnormalities (T_max_ ≥ 4 s ≥ 10 mL) showed no significant difference in these variables between the two groups except in history of hypertension (χ^2^ = 5.22; *p* = 0.022). Surprisingly, there was no significant difference in the baseline ABCD2 score between these two groups, ABCD2 score has a strong predictive value of early neurological deterioration. Patients with atrial fibrillation tended to have a larger volume of lesions on PW images (8% compared with 0% of patients with no PWI abnormalities, *p* = 0.094). Patients with focal perfusion abnormalities tended to show more severe stenosis of responsible vessels (*p* = 0.056).

### Prediction of MRI Perfusion Abnormality

In univariate binary logistic regression analysis, history of hypertension (*p* = 0.028, OR = 4.15, 95% CI = 1.17–14.69) was independently associated with MR perfusion deficit. Stenosis (50%) (*p* = 0.065, OR = 3.53, 95% CI = 0.93–13.47) and systolic blood pressure (sBP) (*p* = 0.198, OR = 1.02, 95% CI = 0.99–1.05) on admission tended to be related to perfusion abnormality after a TIA. ABCD2 score (*p* = 0.959, OR = 0.99, 95% CI = 0.70–1.41) was not associated with perfusion abnormality (Table 2). Multivariate regression modeling was performed for predictors with *p* < 0.20.

**Table 2:** Possible predictors of T_max_ ≥ 4 s ≥ 10 ml in patients with TIA (univariate Binary logistic regression analysis).

	**OR**	**95% CI**	***P***
History of hypertension	4.15	1.17–14.69	0.022
Stenosis (50%)	3.53	0.93–13.47	0.056
ABCD2 score	0.99	0.70–1.41	0.959

*CI, confidence interval; OR, Odds ratio*.

*ABCD2: age 60 (1 point), SBP 140 or DBP 90 mm Hg (1 point), clinical features as unilateral weakness (2 points), or speech impairment without weakness (1 point), symptom duration 60 min (2 points), or 10–59 min (1 point), diabetes (1 point)*.

The multivariate logistic regression of associations between history of hypertension, stenosis (50%), sBP, and MR perfusion abnormality was shown in Table 3. It was clear that patients with a history of hypertension had a significantly higher risk of PWI abnormality (T_max_ ≥ 4 s ≥ 10 mL) after a TIA. After adjusting potential confounding factors (age, sex, ABCD2), the odds ratios were 3.89 (95% CI, 1.08–13.96, *p* = 0.037, model 1), 4.33 (95% CI, 1.20–15.65, *p* = 0.025, model 2), and 4.11 (95% CI, 1.12–15.11, *p* = 0.033, model 3), respectively. Stenosis (50%) and sBP on admission were not independently associated with perfusion abnormality after adjusting potential confounders.

**Table 3:** Factors independently associated with T_max_ ≥ 4 s ≥10 ml in patients after TIA.

**Risk factors**	**Model 1**		**Model 2**		**Model 3**	
	**Adusted OR (95% CI)**	***P* value**	**Adusted OR (95% CI)**	***P* value**	**Adusted OR (95% CI)**	***P* value**
History of hypertension	3.89 (1.08–13.96)	0.037	4.33 (1.20–15.65)	0.025	4.11 (1.12–15.11)	0.033
Stenosis (50%)	3.56 (0.92–13.79)	0.067	3.65 (0.94–14.27)	0.063	3.60 (0.91–14.20)	0.067

*CI, confidence interval; OR, Odds ratio; sBP, Systolic blood pressure at admission*.

*ABCD2: age 60 (1 point), SBP 140, or DBP 90 mm Hg (1 point), clinical features as unilateral weakness (2 points) or speech impairment without weakness (1 point), symptom duration 60 min (2 points), or 10–59 min (1 point), diabetes (1 point)*.

*Model 1: adjusted for age and sex*.

*Model 2: adjusted for ABCD2*.

*Model 3: adjusted for age, sex, and ABCD2*.

## Discussion

To the best of our knowledge, this is the first report that presented the prevalence and clinical risk factors for MRI perfusion abnormality in TIA patients of a Chinese population. The prevalence of MR perfusion T_max_ ≥ 4 s ≥ 10 mL was 42.4% (25/59). Meanwhile, we found that among Chinese patients with acute TIA, history of hypertension is an independent factor associated with MR perfusion abnormality (T_max_ ≥ 4 s ≥ 10 mL).

### Prevalence of MR Perfusion Abnormality

Our study showed a 72.9% (43/59) prevalence of MR perfusion (T_max_ ≥ 4 s > 0 mL) in patients with DWI-negative TIA and 42.4% (25/59) (T_max_ ≥ 4 s ≥ 10 mL) had an acute focal PWI lesion without showing a DWI lesion, which is similar to the previous report in Canada which showed a prevalence of 42% (57/137) (13), but higher than the prevalence of 25% (16/64) in South Korea (15) and 23% (57/137) in the United States (14). There are a few possible reasons for the higher prevalence. Firstly, the variability of findings in these studies is likely due to the inconsistent definition of perfusion. A study reported that a regional PWI lesion was detected on time-to-peak (TTP) and Mean transit time (MTT) maps, which were produced by the standard software bound to the scanner (15). Another study showed that a focal perfusion abnormality was identified on either time to maximum of the residue function (T_max_) or Cerebral blood flow (CBF) maps (14). In the present study, T_max_ ≥ 4 s was used to define the regional perfusion abnormality. Secondly, different algorithms used for discrete platforms might be responsible for the discrepancy. Focal perfusion abnormalities were evaluated independently by two observers in some studies (14, 15) or PWI source images were analyzed by a customized Matlab 7.4 (The Mathworks) software (13). However, in this present study we used RAPID to calculate the volume of perfusion. Therefore, the prevalence of MR perfusion abnormality in the present study is higher than that of previous reports. Thirdly, participants in the present study were all inpatients of our stroke center, who were more likely to have perfusion lesions than outpatients because their conditions were more serious.

In the present study, 25 of 59 patients had T_max_ ≥ 4 s ≥ 10 mL. T_max_ delay threshold 4 s seems to be optimal for early assessment of critically hypoperfused tissue (21). T_max_ volume is a good predictor for clinical outcome in MCA occlusions (22). The threshold (T_max_ ≥ 4 s) at a volume of 10 mL is optimal for predicting infarct growth with the maximal sensitivity and specificity (13).

### Risk Factors Associated With MR Perfusion Abnormality

There are multiple possible clinical risk factors for MR perfusion abnormality in the context of TIA. 67.8% of the 59 TIA patients included in this study had a history of hypertension, which is similar to that of previous studies (14, 23, 24). In the present study, 84% of 25 patients with T_max_ ≥ 4 s ≥ 10 mL after TIA onset had a history of hypertension. We found that the increased prevalence of MR perfusion lesions occurred in patients with a history of hypertension, which was further confirmed in the stepwise multiple logistic regression analysis, suggesting that history of hypertension is an independent risk factor for MR perfusion abnormality in patients with TIA. A previous study showed that hypertension could lead to morphological impairment of the cerebral microvasculature, blood-brain barrier disruption, and neuroinflammation (25). Previous findings suggest that acute PWI lesions may be due to a persistent microvascular injury that results in hypoperfusion (15, 26). However, we found that sBP at admission is not a stronger predictor of MR perfusion abnormality after TIA than a history of treated hypertension, which is inconsistent with previous reports on Western populations (23, 24). In their reports, elevated SBP at presentation is more predictive of stroke after a TIA than a history of hypertension (23, 24). There are a couple of possible reasons for this discrepancy. Firstly, Median sBP at admission was measured 2 days after the acute TIA period (>24 h after symptom onset), therefore, it is less likely to reflect the real sBP when TIA occurred and therefore, less predictive for poor short-term prognosis (27). Secondly, the fluctuation of sBP (130–150 mmHg) in the early course of TIA is minimal, which is not associated with poor 90-day survival (28). Together, our findings suggest that the history of hypertension, but not sBP at admission, is significantly associated with local PWI lesions after a TIA.

In subset analysis of our participants with MR perfusion abnormalities, one-thirds (8/25) of the patients had evidence of proximal artery stenosis or occlusion, which is consistent with previous reports (11, 14, 29). In our study, proximal artery stenosis (>50%) tended to have a larger PW lesion on MRI scans (adjusted OR = 3.60, 95% CI (0.91–14.20), *p* = 0.067). This finding suggests the added diagnostic value of MR perfusion imaging with MRA for detection of hemodynamic abnormality within the microvasculature (13, 30). In the present study, the widely used ABCD2 score was not associated with perfusion deficit, which is similar to what was reported by a previous study (30). The possible explanation for this might be that ABCD2 score is based on patients' clinical factors and does not include information about brain hemodynamics.

This study has a number of limitations. Firstly, it is a cross-sectional study design and cannot demonstrate direct causality between MR perfusion and the risk factors in subjects with TIA. A longitudinal design can help to investigate the direct causality of MR perfusion in future studies. Secondly, we had a relatively small sample size, possibly introducing unknown patient selection bias. Therefore, a large sample size would be optimal for confirming our findings. Thirdly, all patients were recruited from inpatients admitted to one local hospital. Hence, conclusions and observations should be treated with caution. However, our hospital is the first and the only one that can use RAPID to calculate the volume of T_max_ ≥ 4 s within the first 7 days after a TIA attack in China. Fourthly, the present study lacks imaging and clinical follow-up. It is unknown whether perfusion abnormalities observed were reversible or progressed to infarction after initial imaging. Therefore, the findings in this study should be considered as preliminary and should be confirmed in future studies. Fifthly, in this study we used T_max_ ≥ 4 s for defining perfusion deficits (21), and volume of T_max_ ≥ 4 s > 10 mL for defining perfusion abnormality (13). Although our method is based on a previous study, whether this method has better accuracy and applicability needs further prospective, large-scale studies to verify.

In conclusion, history of hypertension is a strong predictor of focal perfusion abnormality calculated by RAPID on T_max_ maps in DWI-negative TIA patients. However, further prospective studies including a larger number of patients are needed to confirm this finding.

## Data Availability

All datasets generated for this study are included in the manuscript and/or the supplementary files.

## Ethics Statement

Ethical approval for this study was obtained from Human Research Ethics Committee of Shanghai Fourth People's Hospital Affiliated to Tongji University School of Medicine. Written informed consent was obtained from all subjects.

## Author Contributions

YW, HL, and SW contributed to design and conceptualization of the study, data collection, analysis, and interpretation of the data, and drafting of the original manuscript. YL contributed to data collection and revision of the manuscript. YZ contributed to data collection and revision of the manuscript. SW, YB, and LJ contributed to data interpretation and revision of the manuscript.

### Conflict of Interest Statement

The authors declare that the research was conducted in the absence of any commercial or financial relationships that could be construed as a potential conflict of interest.
